# Hwb learning platform: data insights into learning and teaching continuity throughout the COVID-19 pandemic.

**DOI:** 10.23889/ijpds.v7i3.2080

**Published:** 2022-08-25

**Authors:** Alexandra Sandu

**Affiliations:** 1 Cardiff University - Wales Institute of Social and Economic Research and Data

The COVID-19 pandemic has prompted everyone to adapt and find a solution to face-to-face contact, including the educational community, which had to suspend face-to-face teaching in order to prevent the spread of the new virus. Wales, confronted with the need to close schools to avoid the spread of the virus, did so in March 2020, shifting the majority of its delivery online. Indeed, Wales’ Hwb Digital Learning Platform was perfectly poised as a resource to address this unique challenge. Furthermore, because it provides monthly usage statistics across the system, it was a valuable resource for monitoring the transition to online digital teaching and learning and, more importantly, identifying any variations or inequalities in usage. Linking administrative datasets containing information about the schools and Hwb usage (logins) for the academic year 2019/2020, this research aims to explore the continuity of learning and teaching in Wales using statistical methods, by highlighting patterns and changes in the Hwb Learning Platform usage during the lockdown in Wales, with an emphasis on the first school closure owning to the COVID-19 pandemic, as well as the digital learning inequalities that have emerged during the same timeframe. The findings show that during the lockdown, pupils and staff are more engaged with Hwb, with a peak in April 2020, however, engagement varies significantly not only across school sectors but also between Consortia and within them.

